# Tryptophan Substitution in CJ-15,208 (*cyclo*[Phe-D-Pro-Phe-Trp]) Introduces δ-Opioid Receptor Antagonism, Preventing Antinociceptive Tolerance and Stress-Induced Reinstatement of Extinguished Cocaine-Conditioned Place Preference

**DOI:** 10.3390/ph16091218

**Published:** 2023-08-29

**Authors:** Kristen H. Scherrer, Shainnel O. Eans, Jessica M. Medina, Sanjeewa N. Senadheera, Tanvir Khaliq, Thomas F. Murray, Jay P. McLaughlin, Jane V. Aldrich

**Affiliations:** 1 Department of Pharmacodynamics, The University of Florida, Gainesville, FL 32610, USA; kristen_scherrer@med.unc.edu (K.H.S.); shaieans@ufl.edu (S.O.E.); jjjessicamariemedina@yahoo.com (J.M.M.); 2 Department of Medicinal Chemistry, The University of Kansas, Lawrence, KS 66045, USA; nilendrasns@yahoo.com (S.N.S.); tanvir.khaliq@sdstate.edu (T.K.); 3 Department of Medicinal Chemistry, The University of Florida, Gainesville, FL 32610, USA; 4 Department of Pharmacology and Neuroscience, Creighton University School of Medicine, Omaha, NE 68178, USA; tfmurray@creighton.edu

**Keywords:** opioid peptides, macrocyclic tetrapeptides, multifunctional ligands, structure–activity relationships, metabolic stability, kappa opioid receptor, delta opioid receptor, analgesics, opioid liabilities

## Abstract

The macrocyclic tetrapeptide CJ-15,208 (*cyclo*[Phe-D-Pro-Phe-Trp]) and its D-Trp isomer exhibit kappa opioid receptor (KOR) antagonism which prevents stress-induced reinstatement of extinguished cocaine-conditioned place preference. Here, we evaluated the effects of substitution of Trp and D-Trp on the peptides’ opioid activity, antinociceptive tolerance, and the ability to prevent relapse to extinguished drug-CPP. Six analogs were synthesized using a combination of solid-phase peptide synthesis and cyclization in solution. The analogs were evaluated in vitro for opioid receptor affinity in radioligand competition binding assays, efficacy in the [^35^S]GTPγS assay, metabolic stability in mouse liver microsomes, and for opioid activity and selectivity in vivo in the mouse 55 °C warm-water tail-withdrawal assay. Potential liabilities of locomotor impairment, respiratory depression, acute tolerance, and conditioned place preference (CPP) were also assessed in vivo, and the ameliorating effect of analogs on the reinstatement of extinguished cocaine-place preference was assessed. Substitutions of other D-amino acids for D-Trp did not affect (or in one case increased) KOR affinity, while two of the three substitutions of an L-amino acid for Trp decreased KOR affinity. In contrast, all but one substitution increased mu opioid receptor (MOR) affinity in vitro. The metabolic stabilities of the analogs were similar to those of their respective parent peptides, with analogs containing a D-amino acid being much more rapidly metabolized than those containing an L-amino acid in this position. In vivo, CJ-15,208 analogs demonstrated antinociception, although potencies varied over an 80-fold range and the mediating opioid receptors differed by substitution. KOR antagonism was lost for all but the D-benzothienylalanine analog, and the 2′-naphthylalanine analog instead demonstrated significant delta opioid receptor (DOR) antagonism. Introduction of DOR antagonism coincided with reduced acute opioid antinociceptive tolerance and prevented stress-induced reinstatement of extinguished cocaine-CPP.

## 1. Introduction

The majority of opioid ligands used clinically for the treatment of pain are mu opioid receptor (MOR) agonists, although agonists of the kappa (KOR) and delta (DOR) opioid receptors also produce analgesia. Opioid analgesics remain efficacious therapeutics for moderate to severe pain, but their use is limited by clinical liabilities, including concurrent substance abuse disorders [1]. MOR-selective agonists are reinforcing, and produce analgesic tolerance and respiratory depression [2]. In contrast, KOR-selective agonists produce dysphoria, sedation, and psychotomimetic effects [3], while DOR-selective agonists can induce seizure activity [4]. Multifunctional opioids, ligands with mixed agonist and/or antagonist activity at one or more opioid receptor, have demonstrated potent antinociception, possibly due to synergistic effects [5], or receptor–receptor modulation of signaling, trafficking, and expression between specific opioid receptors, notably the MOR and DOR (for reviews, see references [2,3]). For instance, reports demonstrate that co-administration of DOR agonists increases morphine-induced antinociception [4,5], while DOR antagonists decrease morphine-induced antinociceptive tolerance [6,7,8]. Consistent with this, bifunctional MOR-agonist/DOR-antagonist ligands that target the MOR and DOR, and/or MOR–DOR heteromers, are being explored as possible therapeutic agents and as tools to examine MOR–DOR interactions (for reviews, see references [3,9]). Several have demonstrated analgesic potency equivalent to or greater than morphine, along with a decreased incidence of tolerance in rodents [10,11,12,13,14,15,16,17,18]. Moreover, bifunctional MOR-agonist/DOR-antagonists have been reported to be devoid of reward-seeking behaviors when administered alone, and treatment with MOR-agonist/DOR-antagonists did not induce reinstatement of extinguished morphine-conditioned place preference (CPP) in mice [12]. Early MOR-agonist/DOR-antagonists were also effective in suppressing morphine withdrawal in rhesus macaques [19]. However, the effects of bifunctional MOR-agonist/DOR-antagonists on cocaine-seeking behavior have yet to be examined.

**Figure 1:**
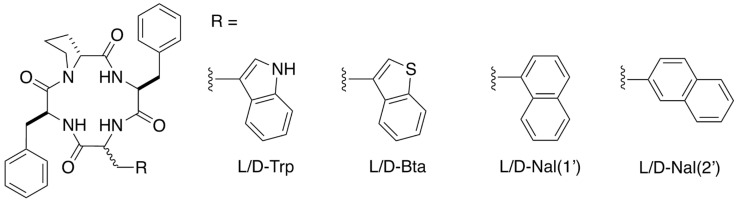
Structures of CJ-15,208, [D-Trp]CJ-15,208 and their analogs containing substitutions for the tryptophan residue. The residues are arbitrarily numbered starting with the Phe residue coupled to the D-Pro residue so that the tryptophan residue is in position 4.

Multifunctional opioid activity has been observed for the structurally distinct macrocyclic tetrapeptide natural product CJ-15,208 (*cyclo*[Phe-D-Pro-Phe-Trp], Figure 1). While the natural product exhibited mixed, multifunctional activity, with robust antinociception mediated by both the KOR and MOR, followed by short-lasting KOR-selective antagonism after oral administration; in contrast, the d-Trp isomer of CJ-15,208 primarily exhibited KOR antagonism with modest antinociception only at elevated doses [20,21].

In the current study, we explored the effects of substitutions for the tryptophan residues in CJ-15,208 and [d-Trp]CJ-15,208 (*cyclo*[Phe-d-Pro-Phe-d-Trp]) on opioid activity, characterizing receptor affinity and analgesic potency, opioid receptor antagonist properties, metabolic stability, and potential liabilities. While the KOR antagonist activity of the parent peptides was generally lost, [Nal(2′)^4^]CJ-15,208 surprisingly demonstrated mixed MOR-agonist/DOR-antagonist activity, prompting us to hypothesize that it would demonstrate both decreased antinociceptive tolerance in an acute assay and potentially prevent reinstatement of extinguished cocaine-conditioned place preference.

## 2. Results

### 2.1. Design and Synthesis

We explored substitutions for the Trp residue that maintained the 2-ring system of the indole. Benzothienylalanine (Bta, Figure 1) was substituted for Trp to examine the potential contribution of the indole NH to the observed opioid activity of the lead peptides. The regioisomers of naphthylalanine (Nal, Figure 1) with two 6-membered rings were also incorporated into analogs to assess the importance of the 6/5 ring system in the indole of Trp and the influence of the orientation of the distal phenyl ring on the opioid activity profiles of the peptides.

The analogs were synthesized by a combination of solid phase synthesis of the linear precursors followed by cyclization in solution using modifications to our original strategy [20,22] to improve the yields of the macrocyclic tetrapeptides. The peptides were purified by silica gel flash chromatography, which facilitates obtaining larger quantities of the pure peptides for in vivo pharmacological evaluation. The peptides were all obtained in high purity (>97%) and reasonable yields (49–82% from the linear precursors) after purification.

### 2.2. In Vitro Pharmacological Evaluation

The affinities of the analogs were determined in radioligand equilibrium competition binding assays (Table 1). The KOR and MOR affinities of the analogs varied depending upon the substitution incorporated. The KOR affinities of the CJ-15,208 analogs decreased 2- to >10-fold, while the MOR affinities of the Bta^4^ and Nal(1′)^4^ analogs increased 4- to 5-fold compared to the parent macrocyclic peptide. In contrast, both the KOR and MOR affinities of the [D-Trp]CJ-15,208 analogs generally increased relative to the parent peptide. The KOR affinity of the D-Bta^4^ analog was 5-fold higher than [D-Trp]CJ-15,208, while the MOR affinities of the D-Bta^4^ and D-Nal(2′)^4^ were 3- to 4-times higher than the parent peptide. All of the analogs exhibited at best low (micromolar) affinity for DOR, similar to the parent peptides.

Analogs were also evaluated in a [^35^S]GTPγS assay to assess KOR agonist activity. Consistent with the results for CJ-15,208 and [d-Trp]CJ-15,208 [24], the analogs did not exhibit appreciable efficacy for either the KOR or MOR when screened at 10 μM in [^35^S]GTPγS assays.

### 2.3. Metabolic Stability

While they are stable to proteases, macrocyclic peptides are known to undergo metabolism by liver microsomal P_450_ enzymes [23] (Khaliq, manuscript in preparation). Therefore, we examined the metabolic stability of the analogs in mouse liver microsomes. Similar to the results for [D-Trp]CJ-15,208 and CJ-15,208, the analogs with a D-amino acid in place of the tryptophan residue were rapidly metabolized by mouse liver microsomes (t_1/2_ = 7–12 min), while the analogs evaluated containing an L-amino acid were more stable (t_1/2_ = 28 and >60 min for the Bta^4^ and Nal(2′)^4^ analogs, respectively).

### 2.4. In Vivo Pharmacological Evaluation

#### 2.4.1. Antinociception

**Figure 2:**
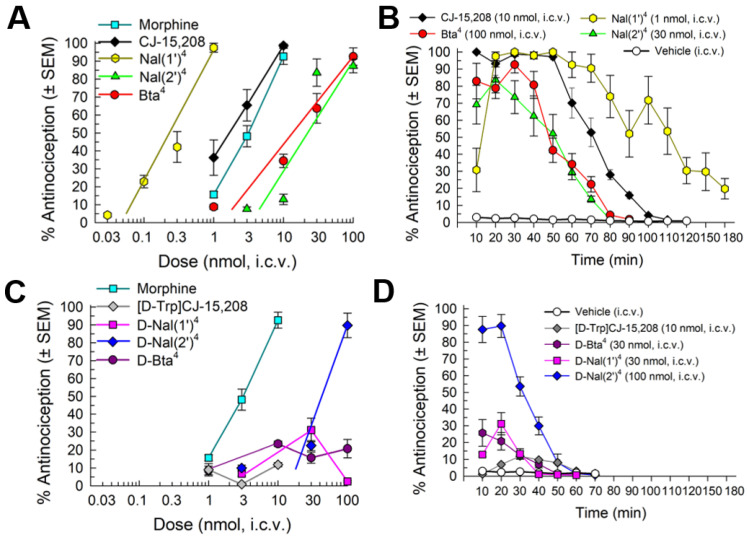
Antinociceptive activity in the 55 °C warm-water tail-withdrawal assay following i.c.v. administration in C57BL/6J mice. Left side: Dose–response of parent compound CJ-15,208 and L-Trp-substituted analogs (**A**) or [D-Trp]CJ-15,208 and D-Trp-substituted analogs (**C**). Morphine is included as a control. All points represent antinociception at peak response, which occurred 20–30 min after administration. Right side: Time-course of antinociception following a maximally efficacious dose of (**B**) CJ-15,208 and L-Trp-substituted analogs or (**D**) [D-Trp]CJ-15,208 and D-Trp-substituted analogs. Points represent average % antinociception ± SEM of 8–10 mice for each set presented.

The analogs were initially evaluated for their antinociceptive activity using the 55 °C warm-water tail-withdrawal assay in C57BL/6J mice following intracerebroventricular (i.c.v.) administration (Figure 2). All of the L-Trp analogs produced dose- (Figure 2A) and time-dependent antinociception (F_(40,356)_ = 13.7; *p* < 0.0001; two-way ANOVA; Figure 2B). Peak antinociception was produced within 20 min after i.c.v. administration for the Nal(1′)^4^ and Nal(2′)^4^ analogs, and at 30 min for the Bta^4^ analog (Figure 2B). The duration of significant antinociception vs. vehicle responses (*p* < 0.05; Dunnett’s post hoc test) varied from 80 min (the Nal(2′)^4^ analog) to 90 min (the Bta^4^ analog), with the Nal(1′)^4^ analog exhibiting significant elevation of tail-withdrawal latency up to 180 min (*p* = 0.008, Student’s *t*-test). In contrast, although the d-Trp analogs also produced significant time-dependent antinociception (F_(20,175)_ = 29.2, *p* < 0.0001, two-way RM ANOVA; Figure 2D), with the exception of the D-Nal(2′)^4^ analog, peak antinociception was much smaller and lasted no more than 40 min (*p* < 0.05, Dunnett’s post hoc test; Figure 2C). Among the d-Trp analogs, only the D-Nal(2′)^4^ analog exhibited full antinociception (Figure 2C), producing approximately 90% antinociception at the highest dose tested (100 nmol) 20 min after administration.

All three analogs of CJ-15,208 along with the D-Nal(2′)^4^ analog produced full antinociception, but with significant differences in potency, demonstrated by a comparison of the shift in ED_50_ values using nonlinear regression modeling (F_(4,176)_ = 104.6; *p* < 0.0001; Table 2). The Bta^4^, Nal(2′)^4^, and D-Nal(2′)^4^ analogs exhibited similar modest potencies (Table 2), while the Nal(1′)^4^ analog was 7.3-fold more potent than the parent compound CJ-15,208. In contrast, the D-Bta^4^ and D-Nal(1′) ^4^ analogs produced less than 35% antinociception at the highest dose tested (30 or 100 nmol, i.c.v.), which is similar to the parent D-Trp peptide. [24]

#### 2.4.2. Opioid Receptor Involvement in Analog Antinociception

**Figure 3:**
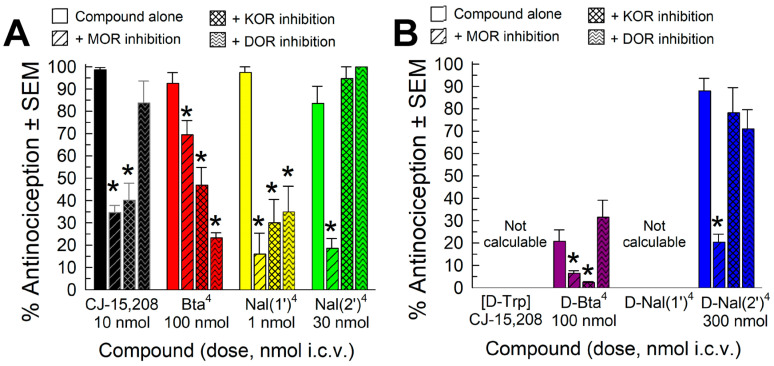
Evaluation of opioid receptor involvement in the antinociceptive activity of the macrocyclic peptides in the 55 °C warm-water tail-withdrawal assay after i.c.v. administration of (**A**) parent compound CJ-15,208 and the Bta^4^, Nal(1′)^4^, and Nal(2′)^4^ analogs and (**B**) the D-Bta^4^ and D-Nal(2′)^4^ analogs that showed significant antinociception. Points represent average % antinociception ± SEM from 8–16 mice for each bar. * significantly different from response of analog alone (*p* < 0.05, one-way ANOVA with Dunnett’s post hoc test).

Contributions of individual opioid receptors to antinociception observed with CJ-15,208 and the analogs of CJ-15,208 and [D-Trp]CJ-15,208 (except for the D-Nal(1′)^4^ analog) were evaluated by testing doses of each compound producing maximum antinociception under conditions selectively inhibiting each type of opioid receptor (Table 2). Except for the Bta^4^ analog, contributions of the MOR and KOR were assessed in knockout (KO) mice for those receptors; DOR contribution to the antinociceptive activity of the analogs was assessed by pretreating mice with naltrindole (20 mg/kg, i.p., −20 min) prior to administration of analog (Figure 3). The Bta^4^ analog was instead tested by pretreating the mice with the selective MOR, KOR, or DOR antagonists β-FNA (5 mg/kg, i.p., −24 h), nor-BNI (10 mg/kg, i.p., −24 h), or naltrindole (20 mg/kg., i.p., −20 min), respectively, prior to administration of the macrocyclic peptide at 100 nmol, i.c.v. (Figure 3A, red bars). Treatment with these antagonists significantly affected antinociception produced by the Bta^4^ analog (F_(3,28)_ = 26.7, *p* < 0.0001, one-way ANOVA), each significantly antagonizing antinociception (*p* ≤ 0.02, Dunnett’s post hoc test), suggesting that all three opioid receptors contributed to the antinociception produced by this analog. While a similar multifunctional profile was displayed in testing with the Nal(1′)^4^ analog (*p* ≤ 0.001, Dunnett’s; yellow bars, Figure 3A), in contrast, the Nal(2′)^4^ and D-Nal(2′)^4^ analogs demonstrated MOR-mediated antinociception, whereas the antinociception produced by [D-Bta^4^]CJ-15,208 was mediated by the MOR and KOR (*p* ≤ 0.05, Dunnett’s post hoc test).

#### 2.4.3. Evaluation of Opioid-Receptor-Selective Antagonist Activity Mediated by the Analog

**Figure 4:**
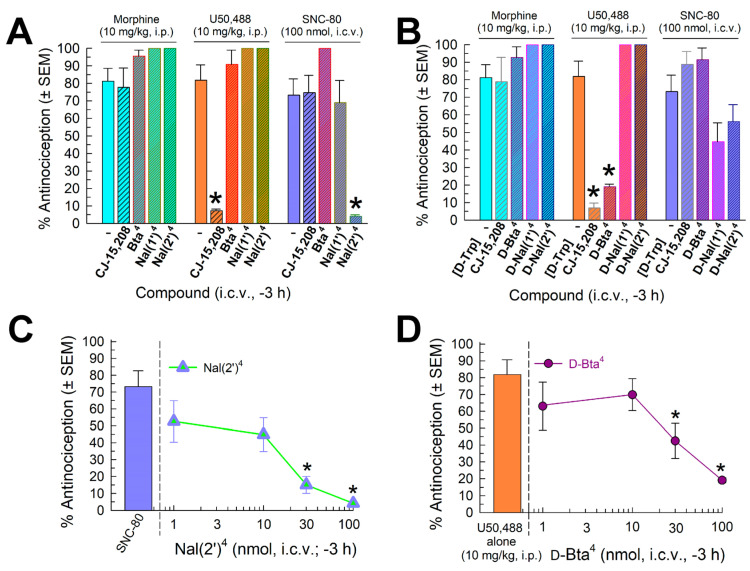
Opioid antagonist activity of the analogs in the 55 °C warm-water tail-withdrawal assay. Mice were pretreated (i.c.v.) with (**A**) parent compound CJ-15,208 (3 nmol) or the Bta^4^ (100 nmol), Nal(1′)^4^ (10 nmol), or Nal(2′)^4^ analog (100 nmol), or (**B**) [D-Trp]CJ-15,208 (3 nmol) or the D-Bta^4^ (100 nmol), D-Nal(1′)^4^ (100 nmol), or D-Nal(2′) analog (300 nmol) 140 min prior to the administration of the MOR-preferring agonist morphine (10 mg/kg, i.p.), the KOR-selective agonist U50,488 (10 mg/kg, i.p.), or the DOR-selective agonist SNC-80 (100 nmol, i.c.v.) to assess their ability to significantly reduce the antinociceptive effect of each opioid agonist. Additional testing examined the antagonist dose–response of (**C**) [Nal(2′)^4^]CJ-15,208-mediated antagonism of SNC-80 or (**D**) [D-Bta^4^]CJ-15,208-mediated antagonism of U50,488. Mean % antinociception ± SEM was determined with 6–14 mice for each bar. * significantly different from response of agonist alone (*p* < 0.05), one-way ANOVA with Dunnett’s multiple comparison post hoc test.

Following the dissipation of any antinociception, the analogs were evaluated for antagonist activity against the MOR-preferring agonist morphine (10 mg/kg, i.p.), the KOR-selective agonist U50,488 (10 mg/kg, i.p.), and the DOR-selective agonist SNC-80 (100 nmol, i.c.v.; Figure 4). Morphine antinociception was not significantly antagonized by compounds with an L-amino acid substitution (F_(4,44)_ = 3.41, *p* = 0.02, one-way ANOVA, but without specific significance in Dunnett’s post-hoc testing; Figure 4A, left bars) or D-amino acid substitutions (F_(4,37)_ = 1.30, *p* = 0.29, one-way ANOVA; Figure 4B, left bars). Moreover, whereas parent peptide CJ-15,208 displayed significant KOR antagonism (F_(4,39)_ = 34.3, *p* < 0.0001, one-way ANOVA with Dunnett’s post-hoc test), substitution of other L-amino acids for Trp abolished this property (Figure 4A, central bars). Likewise, both parent compound [D-Trp]CJ-15,208 and the D-Bta^4^ analog significantly antagonized U50,488-induced antinociception (F_(4,37)_ = 54.8, *p* < 0.0001, one-way ANOVA with Dunnett’s post-hoc test; Figure 4B, central bars); however, substitution with either D-Nal(1′) or D-Nal(2′) for D-Trp eliminated KOR antagonism. Surprisingly, although neither parent peptide antagonized SNC-80, substitution of Nal(2′) for L-Trp in CJ-15,208 conveyed significant DOR antagonism (F_(4,39)_ = 15.4, *p* < 0.0001, one-way ANOVA with Dunnett’s post-hoc test; Figure 4A, rightmost bars); substitution of D-Nal(1′) or D-Nal(2′) in this position in [D-Trp]CJ-15,208 contributed to a generally significant effect of DOR antagonism (F_(4,39)_ = 4.53, *p* = 0.004, one-way ANOVA; Figure 4B, rightmost bars), although this was not significant for any individual analog (*p* = 0.08 or higher, Dunnett’s post hoc test).

### 2.5. In Vivo Assessment of Potential Opioid-Related Liabilities of [Nal(2′)^4^]CJ-15,208

As [Nal(2’)^4^]CJ-15,208 displayed bifunctional MOR agonist/DOR antagonist activity, it was then assessed for potential liabilities produced by standard MOR opioid agonists, specifically antinociceptive tolerance, respiratory depression, hyperlocomotion, and conditioned place preference.

#### 2.5.1. Assessment of Acute Antinociceptive Tolerance Development

[Nal(2’)^4^]CJ-15,208 and morphine were tested in a model of acute antinociceptive tolerance [25] with repeated dosing at 0 and 8 h (1–100 nmol, i.c.v.). The development of acute antinociceptive tolerance was assessed by pretreating with the ED_50_ i.c.v. dose of the test compound, followed 8 h later by treatment with one of a range of graded doses; antinociceptive tolerance was indicated by a significant increase in the ED_50_ value compared to the value observed in naïve animals. As expected, morphine demonstrated acute antinociceptive tolerance, with a significant 7.7-fold rightward shift in the dose–response curve of the second dose administered (F_(2,141)_ = 17.7, *p* < 0.0001, nonlinear regression analysis; Table 3). In contrast, repeated [Nal(2′)^4^]CJ-15,208 treatment significantly reduced the ED_50_ values collected at 8 h vs. 0 h, with a leftward 2.7-fold shift in the dose–response curve of the second dose administered (Table 3).

#### 2.5.2. Evaluation of Respiratory and Spontaneous Locomotor Effects

**Figure 5:**
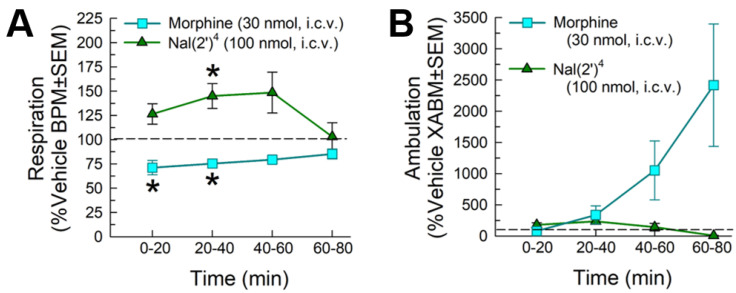
Effects of [Nal(2′)^4^]CJ-15,208 on (**A**) respiration and (**B**) ambulation in C57BL/6J mice tested in the CLAMS/Oxymax system. Respiration and ambulation were monitored after administration of vehicle (i.c.v.), [Nal(2′)^4^]CJ-15,208 (100 nmol, i.c.v.), or the positive control morphine (30 nmol, i.c.v.). Data representing 8–20 mice presented as % vehicle response ± SEM; breaths per minute, BPM (**A**) or ambulation, XAMB (**B**). Dashed lines represent normalized vehicle response. * significantly different from vehicle control response (*p* < 0.05); two-way RM ANOVA with Tukey’s multiple comparison post hoc test.

[Nal(2′)^4^]CJ-15,208 was assessed at the peak antinociceptive dose (100 nmol, i.c.v.) for its effects on respiration rates and spontaneous locomotor activity over an 80 min period using the Comprehensive Laboratory Animal Monitoring System (CLAMS) (Figure 5). As expected, the positive control morphine (30 nmol, i.c.v.) produced significant, time-dependent respiratory depression compared to vehicle (0–40 min; F_(9,168)_ = 2.38, *p* = 0.01, two-way RM ANOVA with Tukey’s multiple comparison post hoc test; Figure 5A). However, treatment with [Nal(2′)^4^]CJ-15,208 did not significantly impair breathing, and in fact statistically increased it briefly from 20 to 40 min compared to vehicle (*p* < 0.05, Tukey’s test; Figure 5A). Similarly, treatment significantly increased ambulation (factor: time x treatment, F_(9,168)_ = 5.19, *p* < 0.0001, two-way RM ANOVA; Figure 5B), but this effect was due to morphine (factor: treatment, F_(1,30)_ = 4.97, *p* = 0.03, two-way RM ANOVA) and not [Nal(2′)^4^]CJ-15,208 (factor: treatment, F_(1,26)_ = 1.40, *p* = 0.25, not significant, two-way RM ANOVA).

#### 2.5.3. Evaluation of Potential Reinforcing or Aversive Properties

**Figure 6:**
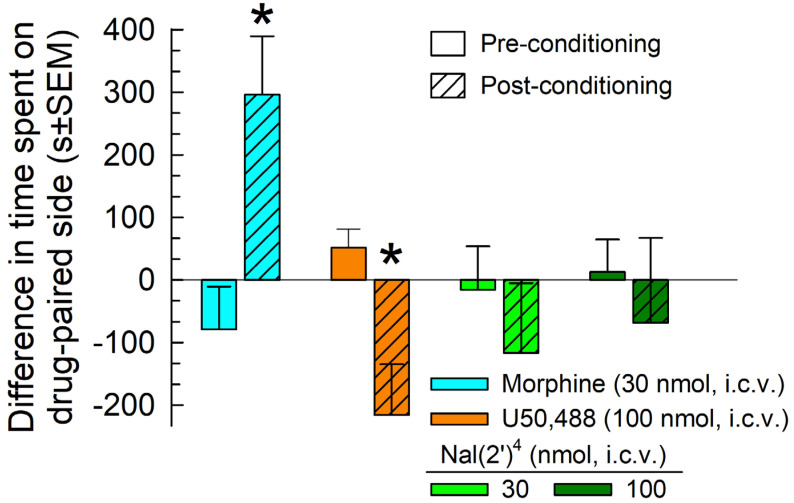
Evaluation of potential rewarding or aversive properties of [Nal(2′)^4^]CJ-15,208. After determination of initial pre-conditioning preferences, C57BL/6J mice were place-conditioned daily for two days with morphine (30 nmol, i.c.v.), U50,488 (100 nmol, i.c.v.), or [Nal(2′)^4^]CJ-15,208 (30 or 100 nmol, i.c.v.) using a counterbalanced design. Data are presented as mean difference in time spent on the drug-paired side ± SEM, with positive and negative values indicating a preference for and avoidance of the drug-paired chamber, respectively. * significantly different from matching pre-conditioning preference (*p* < 0.05), two-way ANOVA with Sidak’s multiple comparison post hoc test. n = 11–22 mice/compound.

[Nal(2′)^4^]CJ-15,208 was assessed for rewarding or aversive properties using a conditioned place preference (CPP) assay. Following a two-day place conditioning paradigm, mice conditioned with morphine (30 nmol, i.c.v.) demonstrated a significant (*p* = 0.03) place preference for the morphine-paired chamber, whereas mice conditioned with U50,488 (100 nmol, i.c.v.) demonstrated a significant (*p* = 0.03) conditioned place avoidance (F_(3,64)_ = 4.89, *p* = 0.004, two-way ANOVA with Sidak’s multiple comparison post hoc test; Figure 6). However, mice place-conditioned with analog [Nal(2′)^4^]CJ-15,208 at either a 30 or 100 nmol, i.c.v. dose demonstrated no significant preference or aversion for their drug-paired chamber (*p* = 0.85 and 0.90, respectively, Sidak’s post hoc test; Figure 6).

#### 2.5.4. Evaluation of [Nal(2′)^4^]CJ-15,208 in the Prevention of Reinstatement of Extinguished Cocaine-Conditioned Place Preference

**Figure 7:**
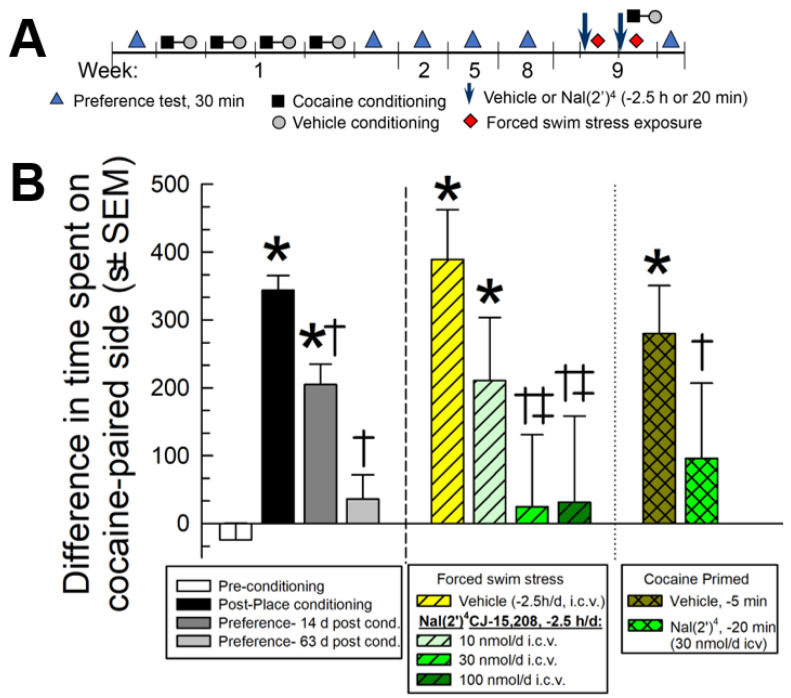
Prevention of stress-induced reinstatement of extinguished cocaine-CPP by [Nal(2′)^4^]CJ-15,208. (**A**) Reinstatement paradigm schematic. (**B**) Following 4 days of cocaine administration (10 mg/kg, s.c. daily), mice exhibited significant preference for the cocaine-paired environment, with extinction occurring 9 weeks later. Mice were then exposed to forced swim stress or another round of cocaine place conditioning, reinstating preference. Pretreatment with [Nal(2′)^4^]CJ-15,208 (10, 30 or 100 nmol, i.c.v.) dose-dependently prevented stress-induced reinstatement of place preference when administered 2.5 h prior to FSS exposure; however, a 30 nmol pretreatment had no significant effect on cocaine-seeking behavior when administered 20 min prior to an additional round of cocaine place conditioning as compared to vehicle-treated animals. Bars represent means of n = 16–31 mice (with 19 vehicle-treated, FSS-exposed mice); cocaine place conditioning data in the left panel represent combined responses of all 132 mice. * significantly different from pre-conditioning place preference response; † significantly different from post-conditioning place preference response; ‡ significantly different from vehicle pretreatment reinstatement of place preference response (*p* < 0.05); one-way ANOVA followed by Tukey’s multiple comparison post hoc test.

[Nal(2′)^4^]CJ-15,208 was tested for its ability to prevent stress- and cocaine-induced reinstatement of extinguished cocaine-seeking behavior (Figure 7). Following 4 days of cocaine-induced place conditioning, mice demonstrated a significant preference for the cocaine-paired chamber (F_(2.53,330.8)_ = 45.9, *p* < 0.0001, one-way RM ANOVA with Tukey’s multiple comparisons post hoc test; Figure 7B, black bar). Extinction of this preference was observed following repeated preference testing over 8 weeks after conditioning (*p* = 0.48 vs. pre-conditioning response, and *p* < 0.0001 vs. post-conditioning response, Tukey’s post hoc; Figure 7B, light grey bar). Mice were then pretreated once daily for two days with vehicle (i.c.v.) or [Nal(2′)^4^]CJ-15,208 (10, 30 or 100 nmol, i.c.v.) and exposed to forced swim stress (FSS) or a single additional cycle of cocaine place conditioning (see reinstatement schematic, Figure 7A). Mice pretreated with vehicle displayed significant reinstatement of drug-seeking behavior after exposure to forced swimming (F_(6,478)_ = 15.2, *p* < 0.0001, one-way ANOVA with Tukey’s post hoc; Figure 7B, yellow central bar). When pretreated 2.5 h prior to FSS, [Nal(2′)^4^]CJ-15,208 dose-dependently prevented stress-induced reinstatement of cocaine-CPP; while a 10 nmol dose proved ineffective (*p* = 0.73, Tukey’s test; Figure 7B, central light green bar), treatment with 30 or 100 nmol significantly decreased reinstatement of cocaine-CPP compared to vehicle-treated mice (*p* = 0.03 and 0.01, respectively, Tukey’s post hoc test; Figure 7B, central green bars). Mice pretreated with vehicle also demonstrated significant reinstatement of cocaine-CPP after exposure to cocaine place conditioning (F_(4,434)_ = 25.2, *p* < 0.0001, one-way ANOVA with Tukey’s post hoc; Figure 7B, brown right bar). While mice given a 20 min pretreatment with [Nal(2′)^4^]CJ-15,208 (30 nmol, i.c.v.) prior to the additional cocaine place conditioning did display a place preference significantly smaller than that of the initial cocaine-CPP (*p* = 0.04; Tukey’s post hoc test), the response did not significantly differ from that of the vehicle-treated, cocaine-place conditioned mice (*p* = 0.39, Tukey’s post hoc test; Figure 7B, rightmost bars).

## 3. Discussion

There were subtle differences in the impact of these amino acid substitutions on opioid receptor affinities and metabolic stability. D-Amino acid substitutions were well tolerated by the KOR, while substitution for L-Trp generally decreased KOR affinity. In contrast, the stereochemistry of this residue only differentially affected the MOR affinities of the L-/D-Nal(2′) analogs. The selectivity of the analogs for the KOR over the MOR was low (K_i_ ratio ≤ 5), except for the D-Bta^4^ derivative, where the 5-fold increase in KOR affinity increased KOR selectivity over the MOR. Similar to the parent peptides, all of the analogs exhibited micromolar affinity for the DOR. The stereochemistry of the residue in this position also affected the metabolic stability; similar to the parent peptides [23], the analogs containing a D-amino acid in this position were rapidly metabolized, while those containing an L-amino acid were more stable.

The antinociceptive activity of the analogs paralleled the results for the respective parent peptides. The peptides containing an L-amino acid at this position all produced maximum antinociception following i.c.v. administration, although the potency varied over an 80-fold range. While the antinociception produced by both the Bta^4^ and Nal(1′)^4^ analogs involved all three receptors, the antinociception produced by [Nal(2′)^4^]CJ-15,208 was mediated only by MOR. In contrast, two of the three analogs containing a D-amino acid exhibited minimal (20–30%) antinociception similar to [D-Trp]CJ-15,208; only [D-Nal(2′)^4^]CJ-15,208 exhibited maximum antinociception, although this was only at high doses.

Only two of the analogs exhibited antagonist activity when tested against standard opioid agonists. Only [D-Bta^4^]CJ-15,208 antagonized antinociception produced by the KOR agonist U50,488, in contrast to the KOR antagonism produced by both of the parent peptides. Unexpectedly, [Nal(2′)^4^]CJ-15,208 significantly antagonized the DOR agonist SNC-80.

Compounds with DOR antagonist activity in addition to MOR agonism have advantages over typical MOR agonists in terms of their safety profile (see references [9,25] for reviews), so we therefore further examined [Nal(2′)^4^]CJ-15,208 for several potential liabilities associated with MOR agonists. Significant tolerance develops to the analgesic effects of MOR agonists such as morphine, requiring higher doses to achieve analgesia. In contrast to morphine, [Nal(2′)^4^]CJ-15,208 did not produce acute tolerance, and its antinociceptive ED_50_ actually decreased significantly in animals pretreated with the peptide. Functional interactions between mu- and delta-opioid receptors (see reference [2]) or the suggested formation of MOR–DOR heterodimers (see reference [3]), as evidenced by a cross-modulation of cellular binding, trafficking, and signaling, may be possible mechanisms for the absence of antinociceptive tolerance. Consistent with this, DOR antagonists decrease morphine-induced tolerance [6,7,8], while morphine-mediated antinociception can be increased by DOR agonists [4,5]. Additionally, i.c.v. pretreatment with DOR-selective antisense oligonucleotides decreased morphine dependence in mice [26], and DOR KO mice continue to demonstrate morphine-mediated antinociception without the development of tolerance [27]. Extending the liability studies, [Nal(2′)^4^]CJ-15,208 notably also did not produce respiratory depression or the hyperlocomotion associated with morphine and other MOR agonists; indeed, the peptide briefly significantly increased respiration. Moreover, the peptide did not exhibit rewarding properties when evaluated in a conditioned place preference assay. None of the opioid macrocyclic tetrapeptides demonstrated gross toxicity in the mice, providing results that are consistent with the observation that peptide-based opioids possess lower toxicity compared to established clinical opioids [28]. More extensive studies can be pursued in the future to evaluate the potential toxicity of promising analogs considered for further development.

The current results with [Nal(2′)]CJ-15,208 represent the first demonstration that a mixed action MOR agonist/DOR antagonist compound is effective at preventing stress-induced reinstatement of extinguished cocaine-conditioned place preference. Such effects are likely mediated by DOR antagonism, given that [Nal(2′)^4^]CJ-15,208 proved effective at doses consistent with those that decreased the antinociceptive efficacy of SNC-80. Consistent with this interpretation, the DOR-selective antagonist SoRI-9409 reduced yohimbine stress-induced reinstatement of alcohol-seeking behavior in rats [29] and was shown earlier to decrease ethanol consumption [30]. Naltrindole also proved effective at blocking cue-induced reinstatement of ethanol-seeking behavior [31,32], further suggesting DOR mediation of reward. DOR antagonists have been shown to both increase and decrease cocaine self-administration in a brain-region-specific manner [33], as well as dose-dependently block cocaine-conditioned place preference and self-administration in rats [34,35,36,37]. The influence of DOR antagonism on reward may stem from changes in the DOR in response to environmental exposure. DORs are redistributed and upregulated following repeated exposure to stress [38], inflammation [39], morphine [40,41], ethanol [42], and ethanol reinstatement [32,43], suggesting that DOR antagonism can be therapeutically effective. Following chronic ethanol exposure, DOR function is increased in several brain areas, including the central nucleus of the amygdala, ventral tegmental area, striatum, and spinal cord [42,44,45,46], and DOR agonists demonstrate greater potency following chronic ethanol exposure [47]. This enhanced DOR function may also be multifaceted, implicated in both DOR-mediated ethanol reward and relapse [48,49], results speculated to be due to DOR activation in the nucleus accumbens and ventral tegmental area increasing dopamine release [49,50]. However, it should be noted that the present finding that DOR antagonists decrease stress-related effects is contrary to evidence showing that the DOR/enkephalin system plays a protective role against stress and mood disorders (for a review, see reference [51]). In general, mice pretreated with a DOR antagonist or mice possessing deleted genes for DOR or enkephalin demonstrated increased anxiety-like behavior in the elevated plus maze, light-dark box, and open field test and increased depression-like behavior in the tail suspension and forced swim tests. In contrast, pretreatment with a DOR agonist decreased the anxiety- and depression-like symptomatology in these assays [51]. The present study did not examine mood, but this might prove a useful subject for future investigations demonstrating the potential application of bifunctional MOR agonist/DOR antagonist ligands in the treatment of substance abuse.

## 4. Materials and Methods

### 4.1. Chemicals

The sources of the reagents, amino acids, solid phase resin, and solvents for peptide synthesis are the same as reported previously [52,53]. Amino acids are the L-isomer unless otherwise specified, and abbreviations for amino acids follow the IUPAC-IUB joint commission of biochemical nomenclature [54]. All other chemicals were obtained from Sigma-Aldrich (St. Louis, MO, USA). Thin layer chromatography was performed on precoated silica gel plates (Sorbent Technologies, glass backed, Atlanta, GA, USA or Whatman, aluminum backed, 250 µm layer, Fisher Scientific, Pittsburg, PA, USA), and flash chromatography was performed on Teledyne RediSep Rf 40–60 µm silica gel cartridges or using standard grade (32–63 μm) silica gel (Sorbent Technologies, Atlanta, GA, USA). 

### 4.2. Instruments

Electrospray ionization mass spectra were acquired on an LCT Premier (Waters Corp., Milford, MA, USA) time-of-flight or an Expression CMS-L (Advion, Ithaca, NY, USA) mass spectrometer. HPLC analysis was performed using an Agilent 1200 HPLC system or a Shimadzu analytical HPLC system.

### 4.3. Peptide Synthesis and Purification

The macrocyclic tetrapeptides were synthesized by a combination of solid phase synthesis of the linear precursors followed by cyclization in solution [52]. The linear peptide precursors Phe-D-Pro-Phe-X were synthesized on a 2-chlorotrityl chloride resin by Fmoc peptide synthesis as previously described [52,53], and the crude linear peptides were cyclized by the general procedure described previously [20,22,23,53]. The crude linear peptide (0.5 equiv, 22 mM in *N*,*N*-dimethylformamide, DMF) was added dropwise (1.4 mL/h) using a syringe pump (KD Scientific, Holliston, MA, USA) to a dilute solution of HATU [2-(1H-7-azabenzotriazol-1-yl)-1,1,3,3-tetramethyluronium hexafluorophosphate] (0.75 equiv, 1.2 mM) and *N,N*-diisopropylethylamine (DIEA, 4 equiv, 6 mM) in DMF. After 15 h, additional HATU (0.75 equiv) was added in one portion to the reaction, and additional linear peptide (0.5 equiv, 22 mM in DMF) was added dropwise (1.2 mL/h) as described above (final peptide concentration 1.1 mM). The reaction was stirred for 6 h at room temperature, and then for an additional 24 h at 37 °C. The solvent was evaporated under reduced pressure, and the crude cyclic peptide isolated as previously described [20,52,53].

The crude peptides were purified by flash silica gel chromatography using a gradient of EtOAc in hexanes. The purified peptides were lyophilized from 20% MeCN in water and analyzed by electrospray ionization mass spectrometry (ESI-MS; see File S1 in Supplementary Materials), thin layer chromatography, and in two different analytical HPLC systems (see Table 4). HPLC analyses were performed on a C18 Vydac 218TP reversed phase column (4.6 × 50 mm, 5 µm) equipped with a guard cartridge at a flow rate of 1 mL/min with detection at 214 nm; the peptides were all >97% pure.

#### 4.3.1. [Bta^4^]CJ-15,208

Following cyclization of the linear peptide (200 mg, 0.33 mmol) the macrocyclic peptide was purified using a gradient of 30 to 100% EtOAc in hexanes over 20 min to give pure [Bta^4^]CJ-15,208 (96 mg, 49% yield) as a white solid following lyophilization.

#### 4.3.2. [Nal(1′)^4^]CJ-15,208

Following cyclization of the linear peptide (200 mg, 0.33 mmol total) the macrocyclic peptide was purified by flash chromatography using a step gradient of 60 to 100% EtOAc (10% increments) in hexanes, followed by a step gradient of 0 to 3% MeOH (1% increments) in EtOAc to yield pure [Nal(1′)^4^]CJ-15,208 (98 mg, 50% yield) as a white solid following lyophilization.

#### 4.3.3. [Nal(2′)^4^]CJ-15,208

Following cyclization of the linear peptide (250 mg, 0.41 mmol total, final concentration 1.7 mM), the macrocyclic peptide was purified using a gradient of 50 to 100% EtOAc in hexanes over 20 min to give pure [Nal(2′)^4^]CJ-15,208 (198 mg, 82% yield) as a white solid following lyophilization.

#### 4.3.4. [D-Bta^4^]CJ-15,208

Following cyclization of the linear peptide (160 mg, 0.27 mmol), the macrocyclic peptide was purified using a gradient of 30 to 100% EtOAc in hexanes over 20 min to give pure [Bta^4^]CJ-15,208 (94 mg, 61% yield) as a white solid following lyophilization.

#### 4.3.5. [D-Nal(1′)^4^]CJ-15,208

Following cyclization of the linear peptide (300 mg, 0.49 mmol total), the macrocyclic peptide was purified using a gradient of 40 to 100% EtOAc in hexanes over 20 min to give pure [D-Nal(1′)^4^]CJ-15,208 (165 mg, 57% yield) as a white solid following lyophilization.

#### 4.3.6. [D-Nal(2′)^4^]CJ-15,208

Following cyclization of the linear peptide (300 mg, 0.49 mmol total), the macrocyclic peptide was purified using a gradient of 30 to 100% EtOAc in hexanes over 20 min to give pure [D-Nal(2′)^4^]CJ-15,208 (194 mg, 67% yield) as a white solid following lyophilization.

### 4.4. Metabolism by Mouse Liver Microsomes

The metabolism of the macrocyclic tetrapeptides by mouse liver microsomes was evaluated as previously described [23], with the amount of peptide remaining determined by LC-MS/MS [55] using [D-MeAla^2^]CJ-15,208 [53] as the internal standard.

### 4.5. In Vitro Pharmacological Evaluation

Opioid receptor affinities were determined by equilibrium radioligand binding assays as previously described [24,53,56] with membranes from Chinese hamster ovary (CHO) cells stably expressing rat KOR, rat MOR, or mouse DOR using the radioligands [^3^H]diprenorphine, [^3^H][D-Ala^2^,N-MePhe^4^,glyol]enkephalin (DAMGO), and [^3^H][D-Pen^2^,D-Pen^5^]enkephalin (DPDPE), respectively. Following determination of IC_50_ values by nonlinear regression using Prism software (GraphPad Software Co., La Jolla, CA, USA), K_i_ values were calculated using the Cheng–Prusoff equation [57]. The results are presented as the mean ± SEM from at least three separate experiments, each performed in triplicate.

The agonist stimulation of [^35^S]GTPγS binding to membranes from CHO cells stably expressing opioid receptors was determined as previously described [24,53]. When screened at 10 µM for efficacy, the peptides exhibited negligible stimulation of GTPγS binding at both the KOR and MOR.

### 4.6. In Vivo Testing

#### 4.6.1. Animals and Drug Administration

Adult male wild-type C57BL/6J mice weighing 20–25 g were obtained from Jackson Labs (Bar Harbor, ME, USA). Additionally, adult male, mu-opioid receptor gene-disrupted “knockout” (MOR KO; B6.129S2-Oprm1tm1Kff/J) and kappa-opioid receptor gene-disrupted “knockout” (KOR KO; B6.129S2-Oprk1tm1Kff/J) mice were obtained from colonies at the University of Florida, established from homozygous breeding progenitor pairs obtained from Jackson Labs. All knockout mice are on the C57BL/6J background for at least 13 generations. All mice were used at an age of 7–11 weeks at the initiation of testing and were group housed in ventilated cages (maximum of five animals per cage) in a temperature-controlled, specific pathogen-free room kept on a 12 h light–dark cycle and cared for in accordance with the National Institute of Health *Guide for the Care and Use of Laboratory Animals*. Food pellets and distilled water were available ad libitum. All results of animal testing are reported in accordance with ARRIVE guidelines [58]. Upon the completion of testing, all mice were euthanized by inhalation of carbon dioxide, followed by cervical dislocation as a secondary measure, as recommended by the American Veterinary Medical Association.

For intracerebroventricular administration, the macrocyclic peptides were dissolved in dimethyl sulfoxide (DMSO), followed by addition of sterile saline (0.9%) so that the final vehicle was 50% DMSO and 50% saline, and the i.c.v. injections were performed as described previously [53]. This concentration of DMSO was not observed to have antinociceptive or behavioral effects in previous use [9,19,21]. All solutions for animal administration were prepared fresh daily.

#### 4.6.2. Antinociceptive Testing

The 55 °C warm-water tail-withdrawal assay was performed in mice as previously described [59], with the latency of the mouse to withdraw its tail from the water taken as the endpoint (a cut-off time of 15 s was used in this assay). Antinociception was calculated according to the following formula: % antinociception = 100 × (test latency − control latency)/(15 − control latency). Tail-withdrawal data points are the means of 8–16 mice, unless otherwise indicated, with SEM shown by error bars.

The opioid receptor involvement in the agonist activity of the macrocyclic peptides was determined by testing in MOR KO and KOR KO mice as utilized previously [60], except for [Nal(1′)]CJ-15,208. The potential agonist activity of [Nal(1′)^4^]CJ-15,208 was instead determined by pretreating mice with a single dose of β-funaltrexamine (β-FNA, 5 mg/kg, i.p.) or nor-BNI (10 mg/kg, i.p.) 24 h in advance of administration of a dose of a macrocyclic peptide. Assessment of DOR involvement in the agonist activity of each macrocyclic peptide was performed by pretreating additional wild-type mice with a single dose of naltrindole (20 mg/kg, i.p.) 20 min in advance of administration of the macrocyclic peptide.

To determine antagonist activity, mice were pretreated with the macrocyclic peptide 140 min prior to the administration of the MOR-preferring agonist morphine (10 mg/kg, i.p.), KOR-selective agonist U50,488 (10 mg/kg, i.p.), or DOR-selective agonist SNC-80 (100 nmol, i.c.v.); at this time, the antinociceptive activity of the analogs had dissipated. Antinociception produced by the established opioid agonists was then measured 40 min after their administration. The dose response of KOR or DOR antagonism produced by selected analogs was further determined by evaluating antinociception after administration of one of three additional i.c.v. doses of the analog.

#### 4.6.3. Acute Antinociceptive Tolerance Determination

A standardized state of tolerance was induced by administration of morphine or test compound at times 0 and 8 h [61,62,63] to quantitatively evaluate development of acute opioid tolerance. This assay was used to efficiently measure the potential of compounds to cause tolerance using a minimum amount of compound while yielding reliable results. Mice were administered an ED_50_ dose (i.c.v.) of test compound in the morning (time = 0) and a second dose (varying between 1–100 nmol, i.c.v.) 8 h later. The degree of tolerance was calculated from the shift in ED_50_ value from the singly to repeatedly treated condition [64]. All compounds were administered i.c.v., with antinociception assessed 30 min after injection of morphine or at 20 min, the time of peak antinociceptive effect of the macrocyclic peptides, as determined in their initial antinociceptive characterization.

#### 4.6.4. Respiration and Ambulation

Respiration rates (in breaths per minute) and animal locomotive activity (as ambulation) were assessed using the Oxymax/CLAMS system (Columbus Instruments, Columbus, OH, USA) as described previously [60,63]. Mice were habituated to their individual sealed housing chambers for 60 min before testing. Mice were administered [Nal(2′)^4^]CJ-15,208 (100 nmol, i.c.v.), morphine (30 nmol, i.c.v.), or vehicle, as indicated, and 5 min later confined to the CLAMS testing chambers. Pressure monitoring within the sealed chambers measured frequency of respiration. Infrared beams located in the floor measured locomotion as number of beam breaks. Respiration and locomotive data were averaged over 20 min periods for 80 min post-injection of the test compound. Data are presented as % vehicle response ± SEM; ambulation, or breaths per minute.

#### 4.6.5. Evaluation of Potential Conditioned Place Preference (CPP) and Conditioned Place Aversion (CPA)

An automated, balanced three-compartment place conditioning apparatus (San Diego Instruments, San Diego, CA, USA) and a 2 day counterbalanced place conditioning design was used, similar to methods previously described [21]. The amount of time subjects spent in each of the three compartments was measured over a 30 min testing period. Prior to place conditioning, an initial preference test was performed in which the animals could freely explore all open compartments; the animals did not demonstrate significant differences in their time spent exploring the outer left vs. right compartments (*p* > 0.05, Student’s *t*-test). For place conditioning, mice were administered 0.9% saline (i.p.) and consistently confined in a randomly assigned outer compartment: half of each group in the right chamber, and half in the left chamber. Four hours later, mice were administered test compound and confined to the opposite compartment for 40 min. To determine if [Nal(2′)^4^]CJ-15,208 (30 or 100 nmol, i.c.v.) produced CPP or CPA, mice were place conditioned in this way for two days, with a final preference test taken on the fourth day, as this has been shown to produce dependable morphine-CPP and U50,488-induced CPA [65]. Additional groups of mice were place conditioned with morphine (30 nmol, i.c.v.) or U50,488 (100 nmol, i.c.v.) as positive controls for CPP and CPA, respectively.

#### 4.6.6. Cocaine-Conditioned Place Preference, Extinction, and Reinstatement Testing

Mice (n = 132) were place conditioned in a counterbalanced design as described above, except with cocaine (10 mg/kg, s.c.), and consistently confined in the randomly assigned outer compartment for 30 min on each of four, rather than two, days (Figure 7A).

*Extinction.* Preference tests were performed twice weekly for eight weeks after the establishment of cocaine-conditioned place preference until extinction was established (see Figure 7A). Extinction is defined as a statistically significant decrease in the time spent in the drug-paired compartment during the extinction trial as compared to the post-conditioning response after the initial 4 days of cocaine place conditioning.

*Reinstatement.* Following extinction, reinstatement of cocaine-CPP was examined after either exposure to forced swim stress or an additional cycle of cocaine place conditioning as described previously [24,66]. Mice were pretreated daily with vehicle (i.c.v.) or [Nal(2′)^4^]CJ-15,208 (10, 30 or 100 nmol, i.c.v.) 2.5 h/d prior to daily forced swim stress in a two day protocol used to produce stress-induced reinstatement of extinguished CPP. Additional mice were administered vehicle or [Nal(2′)^4^]CJ-15,208 (30 nmol, i.c.v.) 20 min prior to cocaine (10 mg/kg, s.c.) for an additional day of place conditioning. On the day following the completion of stress exposure or cocaine place conditioning, mice were tested for place preference to determine possible reinstatement (Figure 7A,B).

### 4.7. Statistical Analysis

All dose–response lines were analyzed by regression, and ED_50_ (effective dose producing 50% antinociception) values and 95% C.I. were determined using individual data points from graded dose–-response curves with Prism 8.0 software (GraphPad, La Jolla, CA, USA). Percent antinociception was used to determine within-group effects and to allow comparison to baseline latency in tail-withdrawal experiments. The statistical significance of differences between ED_50_ values was determined by evaluation of the ED_50_ value shift via nonlinear regression modeling with Prism software. Significant differences in behavioral data were analyzed by ANOVA (one-way or two-way with repeated measures (RM), as appropriate). Significant results were further analyzed with Sidak’s, Tukey’s, or Dunnett’s multiple comparison post hoc tests, as appropriate. Data for conditioned place preference experiments were analyzed by one-way or two-way RM ANOVA, with analyses examining the main effect of conditioned place preference phase (e.g., pre- or post-conditioning) and the interaction of drug pretreatment and/or exposure. All data are presented as mean ± SEM, with significance set at *p* < 0.05.

## 5. Conclusions

KOR antagonism was sensitive to substitution of the L- or D-Trp residue, with only [D-Bta^4^]CJ-15,208 retaining KOR antagonism. Unexpectedly, the novel macrocyclic tetrapeptide [Nal(2′)^4^]CJ-15,208 demonstrated bifunctional MOR agonism and DOR-selective antagonism, producing antinociception in the mouse 55 °C warm-water tail-withdrawal assay without the clinical liabilities associated with standard MOR agonists, namely tolerance, respiratory depression, or psychostimulation, and lacked rewarding properties in a conditioned place preference paradigm. Notably, [Nal(2′)^4^]CJ-15,208 prevented stress-induced reinstatement of extinguished cocaine-conditioned place preference in a dose-dependent manner. Collectively, these findings suggest that [Nal(2′)^4^]CJ-15,208 along with other multifunctional opioid agonist/antagonist compounds are promising candidates for therapeutic development for pain management and/or substance abuse.

## Figures and Tables

**Table 1:** Opioid receptor affinities of the analogs of CJ-15,208 and [d-Trp]CJ-15,208 ^1^.

	K_i_ (nM ± SEM)		
Analog	KOR	MOR	DOR
CJ-15,208 ^2^	27.4 ± 4.6	451 ± 114	1720 ± 350
Bta^4^	51.9 ± 4.8	90.3 ± 38.5	1600 ± 360
Nal(1′)^4^	103 ± 6	110 ± 16	1920 ± 120
Nal(2′)^4^	358 ± 137	583 ± 59	3300 ± 360
[d-Trp]CJ-15,208 ^2^	21.8 ± 4.8	259 ± 29	4190 ± 860
d-Bta^4^	4.29 ± 0.38	84.2 ± 11.8	>10,000
d-Nal(1′)^4^	21.4 ± 0.7	101 ± 25	>10,000 ^3^
d-Nal(2′)^4^	12.3 ± 1.8	63.8 ± 5	>10,000

^1^ Data are the mean K_i_ values ± SEM from at least three experiments except where noted. ^2^ From [23]. ^3^ n = 2.

**Table 2:** Summary of the in vivo opioid antinociceptive activity of the analogs.

	ED_50_ (and 95% C.I. ^1^) Values	
Compound	i.c.v. (nmol)	Receptors Involved
Morphine	2.35 (1.13–5.03)	MOR
CJ-15,208	1.74 (0.62–4.82)	KOR, MOR
Bta^4^	13.2 (9.62–18.2)	KOR, MOR, DOR
Nal(1′)^4^	0.24 (0.19–0.31)	KOR, MOR, DOR
Nal(2′)^4^	19.5 (15.8–24.0)	MOR
[d-Trp]CJ-15,208	~	-
d-Bta^4^	~	KOR, MOR
d-Nal(1′)^4^	~	-
d-Nal(2′)^4^	31.2 (22.5–41.9)	MOR

^1^ Confidence interval. ~ Maximum antinociception not achieved, precluding calculation of an ED_50_ value.

**Table 3:** Comparison of the antinociception ED_50_ (and 95% C.I.) values (nmol) in naïve subjects and again 8 h after a treatment of an ED_50_ dose of the respective compound.

Compound	Naïve ED_50_ (95% C.I.)	ED_50_ (95% C.I.) Pretreated Mice	Fold-Shift, Naïve ED_50_ vs. Second ED_50_
[Nal(2′)^4^]CJ-15,208	19.5 (15.8–24.0)	7.26 * (5.57–9.71)	0.37
Morphine	2.35 (1.13–5.03)	18.1 * (13.7–23.7)	7.70

Dose–response lines analyzed by regression, with ED_50_ (effective dose producing 50% antinociception) values and 95% C.I. determined using individual data points from graded dose–response curves with Prism 8.0 software (GraphPad, La Jolla, CA, USA). The statistical significance of differences between ED_50_ values was determined by evaluation of the ED_50_ value shift via nonlinear regression modeling with Prism software. * significantly different from ED_50_ in naïve mice (*p* < 0.05).

**Table 4:** Analytical data for analogs of CJ-15,208 and [D-Trp]CJ-15,208.

	ESI-MS *m*/*z*		TLC	HPLC	HPLC
Analog	Observed ^1^	Calc ^1^	R_f_ (EtOAc)	System 1 ^2^	System 2 ^3^
Bta^4^	617.3 ^4^	617.2	0.23	24.3	29.4
Nal(1′)^4^	611.3	611.3	0.27 ^5^	15.1 ^6^	27.4 ^7^
Nal(2′)^4^	611.3	611.3	0.18 ^8^	24.6	28.9
D-Bta^4^	617.3 ^4^	617.2	0.67	32.4	37.4
D-Nal(1′)^4^	611.3	611.3	0.66	32.8	37.4
D-Nal(2′)^4^	611.3	611.3	0.68	32.8	36.9

^1^ [M + Na]^+^; ^2^ 15–55% MeCN with 0.1% TFA over 40 min; ^3^ 30–70% MeOH with 0.1% TFA over 40 min; ^4^ [M + H]^+^ 595.3; ^5^ EtOAc:MeOH 98:2; ^6^ 20–85% MeCN with 0.1% TFA over 45 min; ^7^ 30–70% MeOH over 40 min; ^8^ EtOAc:MeOH 97:3.

## Data Availability

Data is contained within the article and supplementary material.

## Supplementary Materials

The following supporting information can be downloaded at: https://www.mdpi.com/article/10.3390/ph16091218/s1, File S1: Mass spectra.

## Abbreviations

ANOVA: analysis of variance; CI, confidence interval; CJ-15,208, *cyclo*[Phe-D-Pro-Phe-Trp]; CLAMS; Comprehensive Lab Animal Monitoring System; CPA, conditioned place aversion; CPP, conditioned place preference; DAMGO, [D-Ala^2^,N-MePhe^4^,glyol]enkephalin; DMSO, dimethyl sulfoxide; DOR, delta opioid receptor; DPDPE, [D-Pen^2^,D-Pen^5^]enkephalin; [D-Trp]CJ-15,208, *cyclo*[Phe-D-Pro-Phe-D-Trp]; FSS, forced swim stress; i.c.v., intracerebroventricular; i.p., intraperitoneal; KOR, kappa opioid receptor; KOR KO, kappa opioid receptor gene-disrupted mice; MOR, mu opioid receptor; MOR KO, mu opioid receptor gene-disrupted mice; MS, morphine sulfate; RM, repeated measures; SNC-80, (+)-4-[(αR)-α-((2S,5R)-4-allyl-2,5-dimethyl-1-piperazinyl)-3-methoxybenzyl]-N,N-diethylbenzamide; U50,488, (±)-*trans*-3,4-dichloro-N-methyl-N-[2-(1-pyrrolidinyl)cyclohexyl]benzeneacetamide. Amino acids are the L-isomer unless otherwise specified; abbreviations for standard amino acids follow IUPAC-IUB Joint Commission of Biochemical Nomenclature [54]
