# Supplementary material for: Tryptophan Substitution in CJ-15,208 (cyclo[Phe-D-Pro-Phe-Trp]) Introduces δ-Opioid Receptor Antagonism, Preventing Antinociceptive Tolerance and Stress-Induced Reinstatement of Extinguished Cocaine-Conditioned Place Preference
Source: Pharmaceuticals (Basel). 2023 Aug 29;16(9):1218. doi: 10.3390/ph16091218 (PMC10535824; doi:10.3390/ph16091218)

Supplemental information:

Tryptophan substitution in CJ-15,208 (*cyclo*[Phe-D-Pro-Phe-Trp) introduces δ-opioid receptor antagonism, preventing antinociceptive tolerance and stress-induced reinstatement of extinguished cocaine-conditioned place preference

Kristen A. Scherrer, Shainnel O. Eans, Jessica M. Medina, Sanjeewa N. Senadheera, Tanvir Khaliq, Thomas F. Murray, Jay P. McLaughlin and Jane V. Aldrich

Mass spectra:

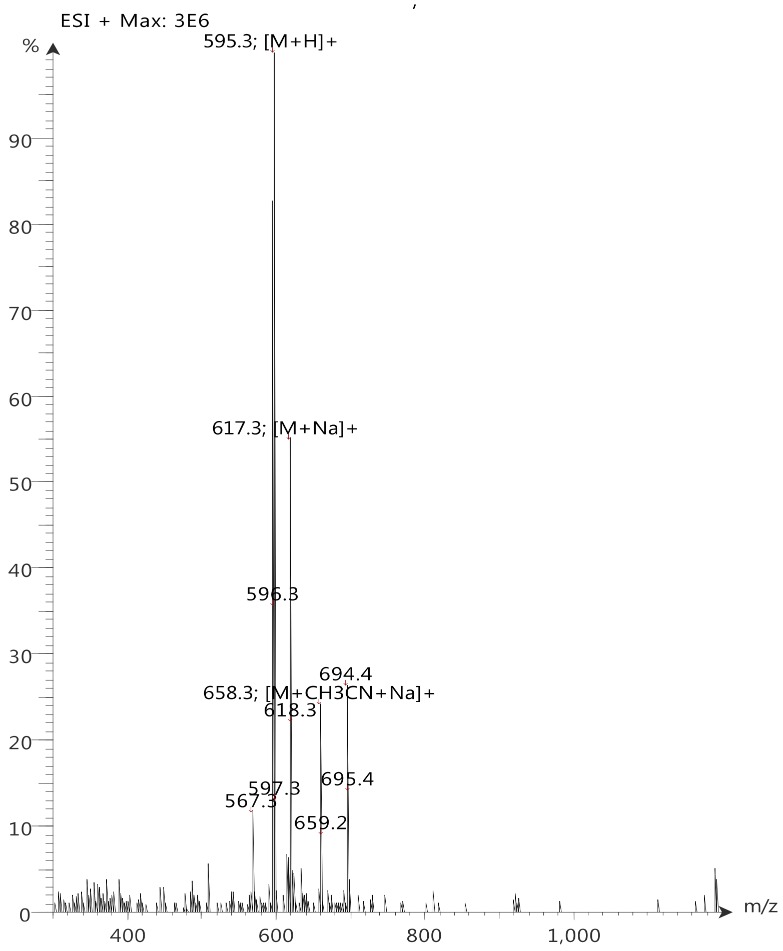

[Nal(1′)^4]CJ-15,208

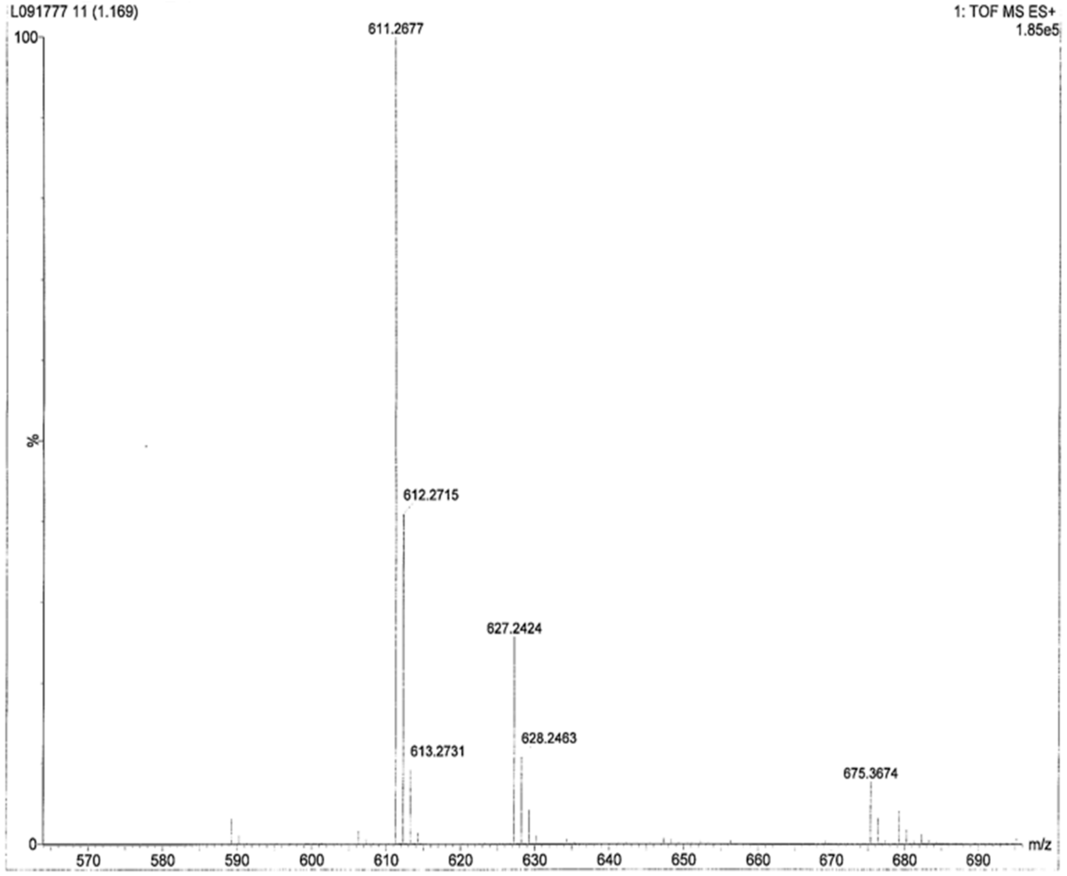

[Nal(2′)[4]]CJ-15,208

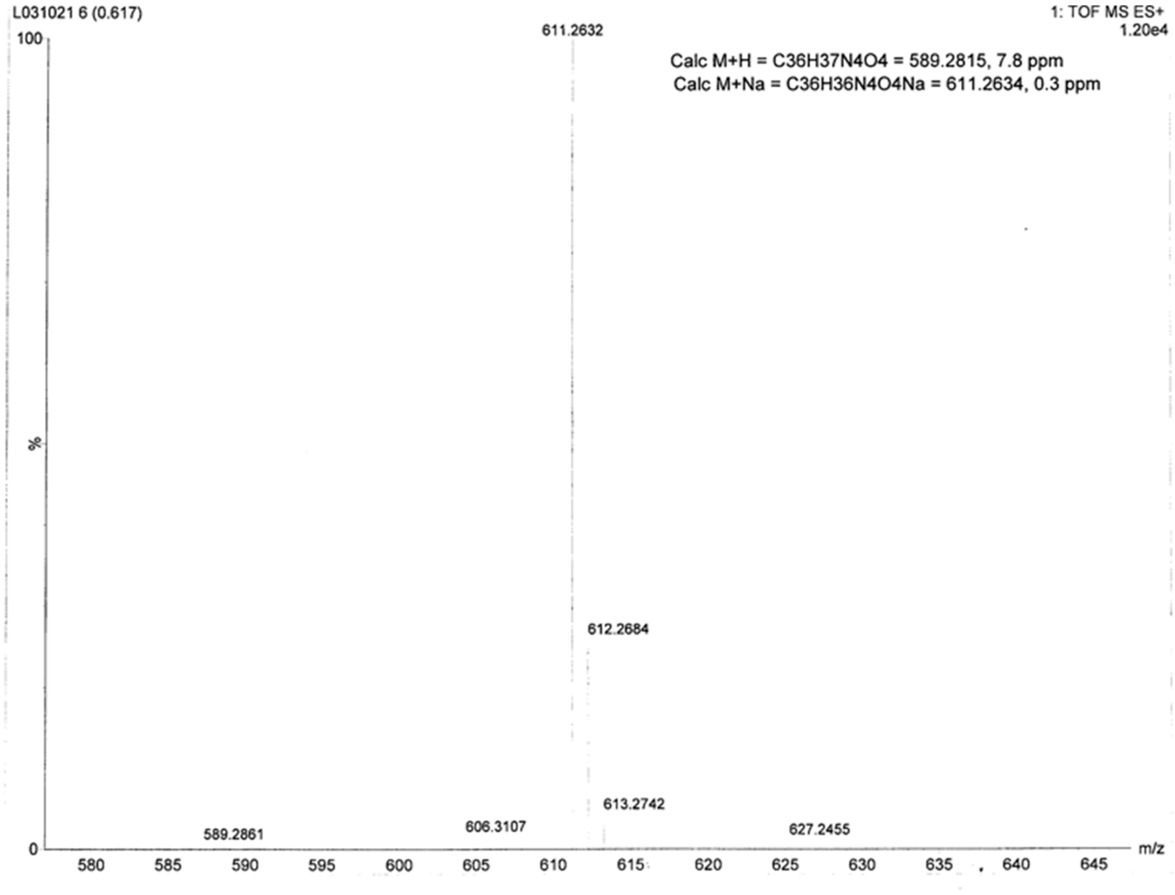

[

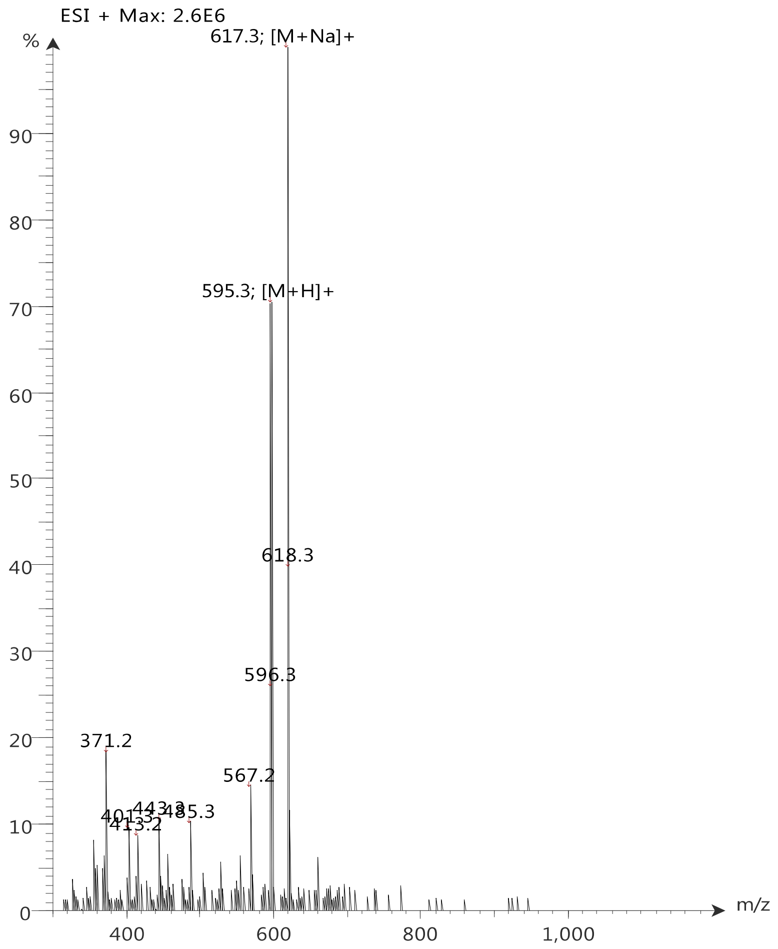

ESI + Max: 2.6E6
%
617.3; [M+Na]+
595.3; [M+H]+
618.3
596.3
371.2
567.2
401.3
443.3
413.2
485.3
0
10
20
30
40
50
60
70
80
90
400
600
800
1,000
m/z

[D-Nal(1′)[4]]CJ-15,208

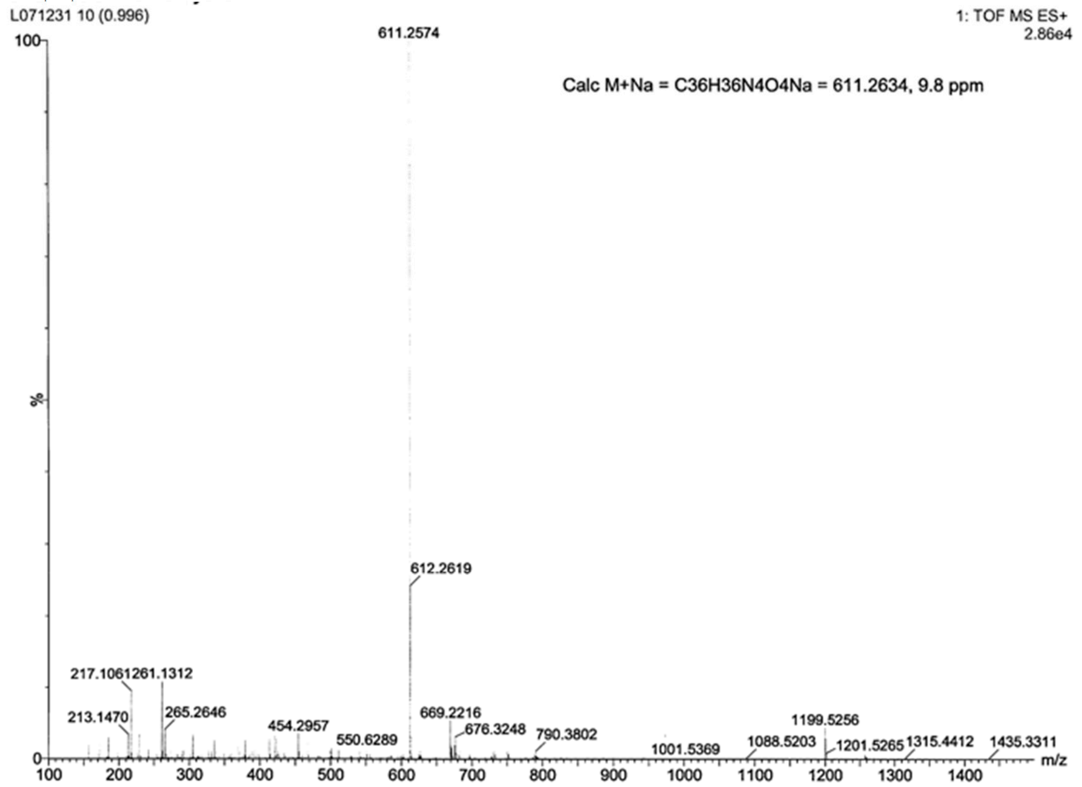

[D-Nal(2′)[4]]CJ-15,208

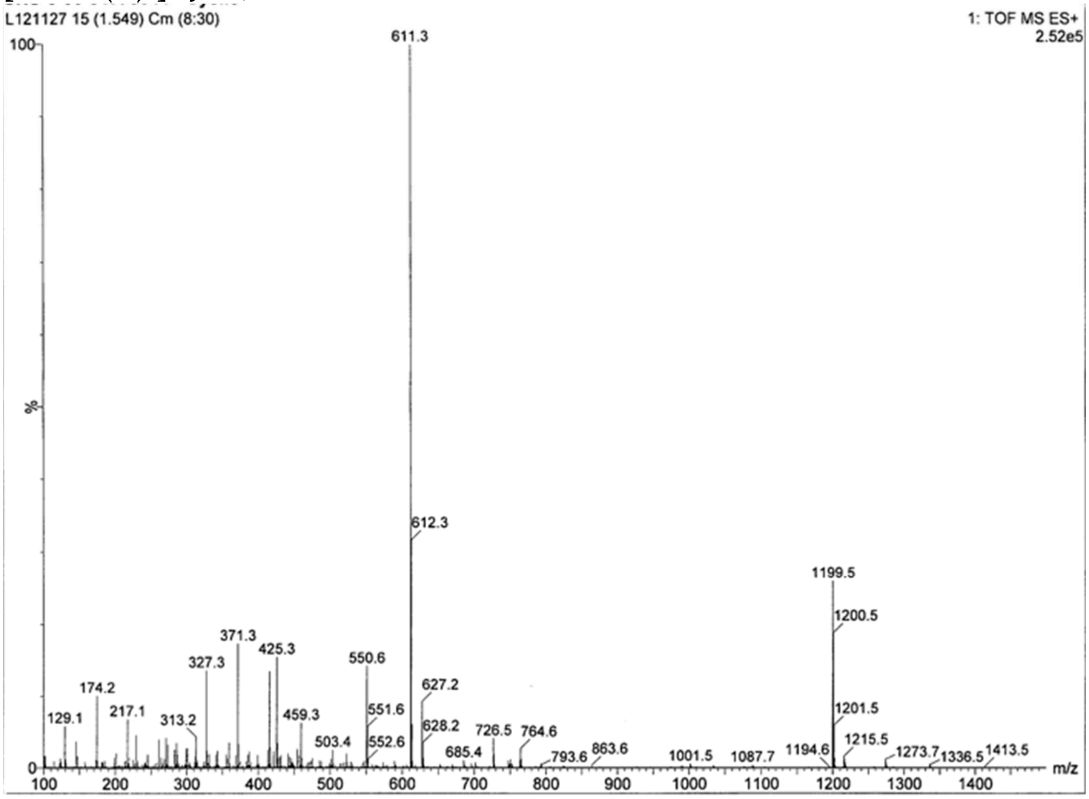

Supplement: Supplementary file 1 [file pharmaceuticals-16-01218-s001.zip › pharmaceuticals-2501552-supplementary.pdf]
